# A hybrid approach to full-scale reconstruction of renal arterial network

**DOI:** 10.1038/s41598-023-34739-y

**Published:** 2023-05-09

**Authors:** Peidi Xu, Niels-Henrik Holstein-Rathlou, Stinne Byrholdt Søgaard, Carsten Gundlach, Charlotte Mehlin Sørensen, Kenny Erleben, Olga Sosnovtseva, Sune Darkner

**Affiliations:** 1 Department of Computer Science, University of Copenhagen, Universitetsparken 1, Copenhagen, 2100 Denmark; 2 Department of Biomedical Sciences, University of Copenhagen, Blegdamsvej 3B, Copenhagen, 2200 Denmark; 3 Department of Physics, Technical University of Denmark, Kongens Lyngby, Copenhagen, 2800 Denmark

**Keywords:** Kidney, Computational models, Data processing, Image processing, Kidney, Kidney, Computer modelling, Computer science

## Abstract

The renal vasculature, acting as a resource distribution network, plays an important role in both the physiology and pathophysiology of the kidney. However, no imaging techniques allow an assessment of the structure and function of the renal vasculature due to limited spatial and temporal resolution. To develop realistic computer simulations of renal function, and to develop new image-based diagnostic methods based on artificial intelligence, it is necessary to have a realistic full-scale model of the renal vasculature. We propose a hybrid framework to build subject-specific models of the renal vascular network by using semi-automated segmentation of large arteries and estimation of cortex area from a micro-CT scan as a starting point, and by adopting the Global Constructive Optimization algorithm for generating smaller vessels. Our results show a close agreement between the reconstructed vasculature and existing anatomical data obtained from a rat kidney with respect to morphometric and hemodynamic parameters.

## Introduction

Computational models of vital organs play an increasing role in the understanding of both normal and diseased organ function. Realistic models of organs require not only a detailed description of the various biochemical and physiological processes but also an accurate representation of the essential anatomy of the organ. In the kidney, the vasculature plays a special role. Not only does it function as a resource distribution network, supplying the individual nephrons with blood and nutrients, but it also constitutes a communication network, allowing contiguous nephrons to interact through electric signaling along the vessels^1^. However, no imaging techniques allow a full reconstruction of the structure of the renal vasculature due to limited spatial resolution.

The primary aim of the present work is the development of a hybrid framework that allows the reconstruction of a realistic model of the full-scale renal vasculature, which matches real anatomical data and can be used in advanced mathematical models of renal function. In addition, real-looking networks that respect the true geometric properties of the organs could allow the simulation of CT scans on the generated vascular trees^2,3^. Such simulated CT scans could help train AI-based models for vascular segmentation on real CT scans, which is one of the long-range aims of the present work. Our approach utilizes existing scans to extract 3D geometrical priors and adopts physiologically based criteria in the construction of the vascular tree^4–6^.

### Biological background

In each organ, the vasculature has a characteristic structure adapted to meet the specific needs of the organ, and a detailed description of the specific vasculature of an organ is necessary for a full understanding of both its physiology and pathophysiology. Nordsletten et al.^7^ provided the hitherto most detailed and quantitative description of the rat renal vasculature. They combined high ($4\, \upmu$m) and low ($20\, \upmu$m) resolution micro-CT images obtained from a vascular cast of a rat kidney. They used the skeletonization method to trace the path of contiguous vessels and then applied the Strahler approach^8^ to sort and interpret the data. Strahler ordering sorts treelike networks by the diameter of the branches according to a bifurcating scheme (see “Strahler ordering”). The principal assumption required for its use is the existence of a diameter-based hierarchy of vessels, ending in the narrowest vessels, i.e., the afferent arterioles supplying the individual nephrons.

Marsh et al.^9^ used micro-CT with $2.5\, \upmu$m resolution to assess the three-dimensional microvascular structure of the rat renal arterial tree. The cast revealed an arterial tree network originating in arcuate arteries, branching as few as twice or as many as six times before reaching a terminal artery that terminated in pairs, triplets, or quadruplets of afferent arterioles. Marsh et al. identified different motives for how afferent arterioles originated from all branch orders of nonterminal arteries or from terminal arteries forming the tops of the arterial trees. Similar branching patterns have been reported by other groups using microdissected trees from four different mammalian species^10–12^.

Postnov et al.^13^ have shown that the pressure drop in a simple bifurcating tree with the vessel dimensions reported by Norsletten et al.^7^ exceeds the value found experimentally. This is expected since in a simple bifurcating tree, afferent arterioles appear only at the terminal branch points of the tree—an assumption that maximizes the hemodynamic resistance between the renal artery and the glomerulus. Postnov et al.^13^ and Marsh et al.^9^ have reported an exponential distribution of the distances between branch points for afferent arterioles across the vascular tree. This distribution, and the possibility to branch from any arterial segment, is the basis for the pressure in the glomerular capillaries being significantly higher, and in a range compatible with normal nephron function, than in a simple bifurcating tree.

The number of nephrons in a kidney in a given species is variable and is thought to play an important role in renal health. Baldelomar et al. estimated nephron numbers from in vivo images and from high-resolution ex vivo images^14^, while Letts et al.^15^ assessed the location of glomeruli in the outer 30% of the cortex, midcortical nephrons (30–60%), and juxtamedullary nephrons of the inner 40% of the cortex. Both studies show high variability in the number and characteristics of nephrons. Taken together, the high variability, both in the structure of the renal vasculature and in the number of nephrons in a given kidney, suggests that a probabilistic-based approach to model nephron-vascular architecture and blood flow dynamics is the right choice.

### Modeling outlook

Three major methods have been described in the literature to construct models of vascular networks. Pure rule-based models^1,13,16^ generate vascular trees analytically from a given root while completely ignoring the spatial structure of the network. The length of each vessel, the radius distribution to its children in a bifurcation, as well as the stopping criteria are all derived from given probability distributions obtained from experimental data. Although the hemodynamics can be simulated without information on the spatial structure, these methods cannot generate real-looking networks and ignore the subject-specific information, and thus cannot be utilized for individual analysis.

The image-based reconstruction methods build 3-D geometric models that capture the high-level structure of an individual’s blood vessels from clinical images^17–19^. These methods involve either a segmentation followed by a centerline extraction or a direct tracking of the blood vessels. Despite advances in deep learning models, learning from very thin structures is still challenging and will suffer from errors due to both vessel merging and discontinuity, resulting in extremely intense manual work afterward. More importantly, in the kidney the vessels at far-surface regions are beyond the experimental resolution, making it impossible to detect the small vessels from an image alone. These small vessels, however, are the ones supplying the individual nephrons and thus have to be resolved in the final model. Therefore, image-based reconstruction alone is unable to provide complete and detailed 3-D vasculatures in the kidney, making biosimulation the only tool available with generative models that can extrapolate modeling to unresolved parts of the kidney.

The angiogenesis-based methods simulate the growth of vasculatures by considering the biological and physiological factors involved in the process such as the size of branching vessels (Murray’s law^5,20^(cf. Eq. (9))) and the hemodynamics in the tree^21^ (cf. Eq. (5)). These algorithms model the vascular tree growing as an optimization problem following the assumption that the network achieves a topological and geometrical structure over the vascularized tissues from hemodynamic principles^22^, e.g., by minimizing the intravascular volume of the tree while ensuring efficient flow. The details of the optimization function are presented in “Physiologically based cost functions”.

There are two main methods to generate the vasculature based on the growing algorithm, namely, Constrained Constructive Optimization (CCO) method proposed by Schreiner and Buxbaum^4^, and its variant Global Constructive Optimization (GCO) proposed by Georg et al.^6^. Both the CCO and GCO algorithms grow the tree inside a pre-defined perfusion territory. In both algorithms, a single tree root location of the blood inlet is chosen manually. In addition, boundary conditions such as terminal radius and flow distributions are imposed to represent physiologic conditions.

These methods are able to generate real-looking vascular structures with both spatial location and connectivity information and have been applied in the liver, heart (left ventricle), and eye^4,6,23^. Recently, Shen et al.^24^ and Ii et al.^25^ incorporated GCO and CCO, respectively, to reconstruct vasculatures in the human brain. Although Cury et al.^22^ has recently applied an adaptive CCO on a prototypical human kidney model, no similar research exists on real renal vasculatures due to the complex non-convex geometry. Moreover, most of the studies produce homogeneous vascular networks that do not account for individual differences, so they cannot be used for individual analysis.

Both CCO and GCO require a convex structure since the connections between any two nodes should not leave the structure. This is one of the reasons why it is challenging to adopt these methods to an organ like the kidney with a complex internal structure (i.e., not convex). Some parts, like the renal pelvis and the pyramids, also pose intrinsic spatial restrictions on vessel construction, which are difficult to model when the tree grows from only a single root node.

Our work follows a similar idea of^24^ by proposing a hybrid way to incorporate subject-specific image-based priors via a semi-automated segmentation of the main (large) arteries and an automatic cortex approximation from the ex-vivo micro-CT scan of a real rat kidney, which both are utilized in the GCO initialization step. In summary, a pre-built arterial tree consisting of the main arteries is extracted from the main artery segmentation, while we also propose a novel approach to sample terminal nodes (glomerulus) from the estimated renal cortex while maximizing the distance between any two neighboring nodes using Poisson disk sampling^26^.

These sampled terminal nodes are then connected to the pre-built vascular tree. Instead of growing from a single root position, the algorithm can now start growing from a pre-built vascular tree and thus will retain subject-specific information in the final generated full-scale vascular tree. At the same time, this procedure avoids violating the intrinsic spatial constraints, because these connections naturally avoid penetrating the middle part of the kidney where the renal medulla resides and do not cross the kidney’s outer boundary, thus making the complex structure piece-wise convex. In contrast to^24^ which proposes forest growth, our algorithm consists of a single tree but with pre-built large branches because of the single inlet of the renal artery. Currently, the image prior starts with a semi-automated segmentation of the vessels, which is time-consuming work. However, recent advances in deep learning are likely in the future to make it possible to automatically segment large vessels given a decent amount of labeled training data, especially in the case of micro-CT with relatively low variation among the images. Apart from the segmentation retrieval and a single user-defined root position, our work is fully automatic, meaning that no software interface is involved in the process. In particular, the leaf nodes sampling and centerline extraction are both implemented in pure Python, unlike^24^ which used BrainSuite for cortex extraction, and^25^ which used Amira for brain hemispheres extraction and skeletonization. All the 3-D software packages, e.g. 3D Slicer^27^ and ParaView^28^ are only used for visualization. This has the advantage that it allows automatization of the process, does not require expert knowledge of the software packages, and provides for better flexibility, e.g., adjustment of parameters to adapt to other organs or image modalities.

Our results show that the structural and functional properties of the reconstructed vascular network are in good agreement with existing anatomical data, e.g., with respect to the radius, length, and pressure distributions^7,29^. We expect the reconstructed full-scale model of the renal vasculature can be utilized to develop realistic computer simulations of renal function or model pathological changes in the kidney, and to develop new image-based diagnostic methods based on artificial intelligence.

## Results

The output from the hybrid framework is a reconstructed renal arterial tree, which begins at the renal artery and ends in the afferent arterioles. In this section, we first present visualizations of the reconstructed tree. In order to quantitatively characterize the tree, we compute the statistics of various variables, e.g., vessel radius and frequency, with respect to the Strahler order (see “Strahler ordering”) of the vessels.

### Model implementation and rendering

**Figure 1:**
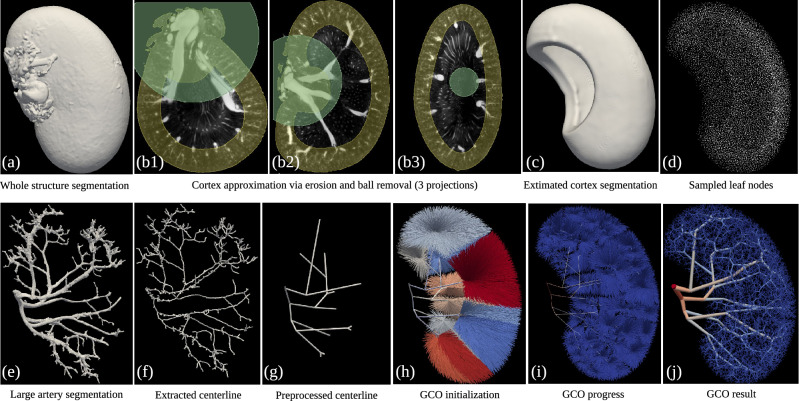
GCO Pipeline, visualized by 3D Slicer^27^ and ParaView^28^. The initial micro-CT scan is used to extract whole structure segmentation (**a**) and large artery segmentation (**e**). Top row: renal cortex (**c**) is approximated by a subtraction of erosion followed by a ball removal (**b**), where the leaf nodes (**d**) are sampled using Poisson disk sampling. Bottom row: extracted centerline (**f**) is pre-processed to pre-build a renal arterial tree consisting of only the first few large arteries (**g**). In GCO initialization (**h**), all the sampled leaf nodes (**d**) are connected to the nearest node in the pre-built tree (**g**) with color indicating the group of leaf nodes that are connected to the same node. Colors in the GCO progress and result (**i**,**j**) indicate the radius of each vessel: from $300\, \upmu$m in renal artery to $10\, \upmu$m in afferent arterioles (AA).

Our hybrid modeling approach to reconstruct a renal vascular network combines semi-automated segmentation of large arteries from micro-CT images and the Global Constructive Optimization algorithm for the generation of smaller microvessels (Fig. 1 and is described in detail in “Methods”). The raw scan has an isotropic voxel size of $22.6\, \upmu$m$^3$. From Table 2 in Nordsletten’s paper^7^, renal arteries with Strahler order from 0 to 2 have a mean radius of 10.08, 13.90, $20.06\, \upmu$m respectively, making it impossible to detect those small arteries from the $22.6\, \upmu$m$^3$ scans. Using our hybrid approach, the small arteries are successfully resolved and connect to the large branches in an anatomically correct manner, producing a full-scale renal arterial tree.

Figure 1j presents a visualization of the generated vascular tree. Each vessel is visualized as a separate cylinder with a thickness corresponding to its radius and color coded by the radius. Although more advanced visualization methods exist^30,31^, this provides a sufficient rendering of the topology of the generated vascular tree. Clearly, if the tree is to be used for more advanced purposes, such as Navier–Stokes-based flow calculations, visualization methods that produce smooth surfaces at junctions and allow the construction of volume meshes will be needed. 3D gif animations with rotation are available at: https://github.com/KidneyAnonymous/RenalArterialTree.

### Morphometric validation

**Figure 2:**
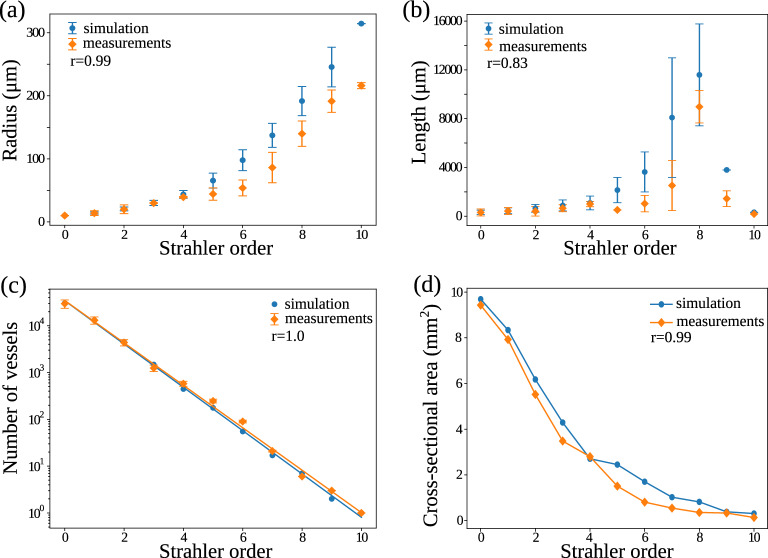
Morphometric features of the generated renal vascular arterial network (simulation) and the experimental data reported in the literature (measurements). In each subfigure, *r* indicates the Pearson correlation coefficient of the mean values with respect to Strahler order. (**a**) Vessel radius vs Strahler order. (**b**) Vessel length vs Strahler order. (**c**) Number of vessels (in log scale) of a particular Strahler order. (**d**) Total cross-sectional area vs Strahler order.

Numerical validation is done by comparing the morphometric properties as shown in Fig. 2, such as the distribution of vessel radius, branch length, and Strahler order in the reconstructed network with data from a real renal arterial tree collected in a rat kidney by Nordsletten et al.^7^. The radius and length of the vessels in a kidney are variable and depend on many factors, including the strain, age, and size of the rat. Although Nordsletten et al.^7^ provided the most detailed and quantitative description of the rat renal vasculature, their collected data are from one kidney in one rat and, therefore, cannot be regarded as a “gold standard”. Instead, it represents one sample from the total population of rats. The purpose of the comparison between the simulated vascular tree and the experimental data is, therefore, not to demonstrate an absolute closeness or identity between the lengths and radii but rather to compare the topology and the distribution of vessel lengths, radii and Strahler orders between them.

In the rat kidney, the radius was found to increase exponentially with the Strahler order of the vessel. The same feature is present in the simulated tree as shown in Fig. 2a . This is contrary to^24^ which shows that the result from GCO and from anatomical data on the brain vasculature both follow a linear increase of radius with Strahler order. This indicates that although the cost function and general process are similar among organs, GCO is able to adjust based on the distinct geometrical features of each organ. The exact values for the radii of the reconstructed vascular tree deviate somewhat from the values reported in^7^, as can be seen in Fig. 2a . This is especially evident for the vessel of the largest Strahler order (the root), which corresponds to the renal artery. In the reconstructed tree, all radii are calculated from the radii of the afferent arterioles using Murray’s law^5,20^ (cf. Eq. (9)). In our initialization process, we assume that the 30K afferent arterioles are derived from the distribution $r_0 \sim {\mathcal {N}}(10.08, 0.14)$ Ref.^7^ (first row of Table 2). Given strict compliance with Murray’s law, the root radius (radius at Strahler order 10) can be computed analytically by $r_{10}=\root 3 \of {\sum _{i=1}^{n=30{,}000} r_{0, i}^3}$ regardless of the branching patterns, which will give a mean value around $\mu (r_{10}) \approx 313.21\ \upmu$m. This number matches our result but deviates from Ref.^7^ (last row of Table 2) where $r_{10} \sim {\mathcal {N}}(216.10, 4.74)$. Similarly, we plot the vessel length for each Strahler order, both from the literature^7^ and from our result, as shown in Fig. 2b . Both the data in the literature and our work show that vessel length has a poor correlation with Strahler order. However, they both show a maximum at order 8, indicating that the large arteries usually are longer, but also that they branch fast when being close to the root, meaning that starting from the renal artery (order 10), vessels only grow to a small length before they branch. This results in a decrease of length with increasing Strahler order from 8 to 10.

Moreover, we plot the number of vessels vs the Strahler order, both from the literature^7^ and from our result, as shown in Fig. 2c . The data from both the literature and our work show an exponential decrease in the vessel numbers vs the Strahler order. As a result, both the vessel numbers from the literature and from our generated tree fit very well to a straight line in log scale.

We further plot the total cross-sectional area vs the Strahler order from our GCO output and the experimental data^7^ in Fig. 2d. Note that the experimental data were extracted from a figure in Ref.^7^ (Fig. 12) and replotted here in Fig. 2d. In agreement with experimental observations, the total cross-sectional area in the generated vascular tree decreases, although the mean radius increases exponentially with Strahler order. Specifically, in both experimental data and our simulated vasculature, the cross-sectional area decreases from a value around 10 mm$^2$, halves after order 2, and keeps decreasing to a value below 0.5 mm$^2$ at the renal artery (order 10). This indicates that the decrease in the number of vessels exceeds the exponential growth of the mean radius with Strahler order. A final interesting property is the Strahler order of the parent vessel of each afferent arteriole, shown in Fig. 4a. Specifically, a parent Strahler order 1 means that afferent arterioles (order 0) branch from terminal arteries (order 1). This case indeed consists of most of the scenarios, but it also demonstrates other possibilities, where afferent arterioles can branch from larger vessels. The parent vessels of the afferent arterioles have Strahler orders from 1 to 8, meaning that in our model, afferent arterioles can branch from any of the larger vessels, except the largest vessels with Strahler order 9 and 10. This characteristic has been shown to be crucial for the ability of the renal vascular tree to supply the glomeruli with blood at a sufficient pressure^1,13^.

Figure 2 also shows the Pearson correlation coefficient of the mean values of the morphometric features between the simulated and experimental data. All the correlations are highly statistically significant and confirm a good agreement between the morphometric features of the two vascular trees.

The above results show that the distribution of e.g., radius and vessel frequency with respect to Strahler order in our generated arterial tree agrees with the literature^7^. Some further properties of the generated vascular tree are noteworthy. The number of Strahler orders in the generated tree was not specified in the optimization process, but notably, the process resulted in a tree with 11 Strahler orders, which match with^7^. Furthermore, given the assumption of around 30 K leaves, our generated tree produces around 51 K vessel segments (edges) in total, with 23 levels (depth) and 11 Strahler orders. On the contrary, if the algorithm simply builds a complete binary tree with perfect symmetries, the total number of vessel segments in the generated tree can be computed by $2 \times 30$ K = 60 K analytically, given the assumption of 30 K leaves. Then, the total number of levels and Strahler orders are identical, and can be computed analytically by $\log _2{60\,{\text {K}}} \approx 16$, which significantly deviates from^7^. This means that asymmetries are correctly inherited in our constructive optimization process, which agrees with the literature that vascular trees are not symmetrically balanced^32–34^.

### Physiological features

**Figure 3:**
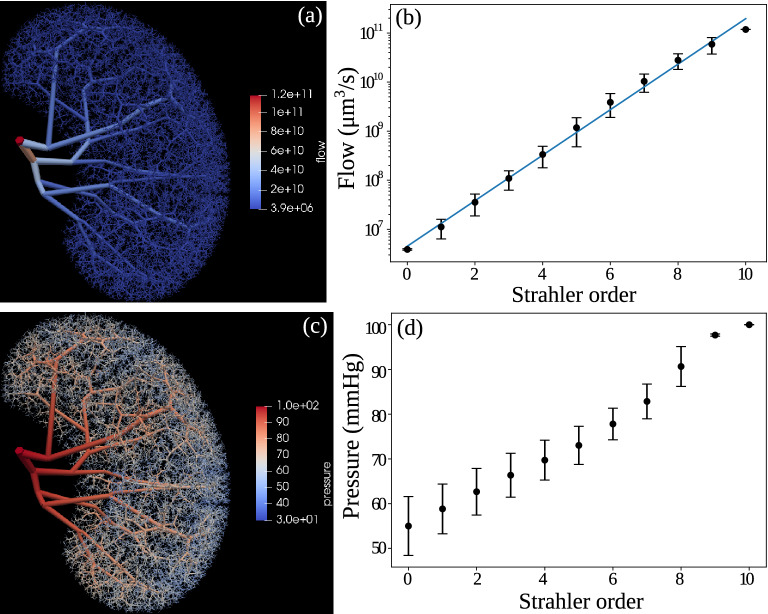
Physiological features of the generated renal vascular network of the rat kidney. (**a**) Visualization of blood flow distributed across the network and (**b**) its distribution vs Strahler order (in log scale) : from $1.2 \times 10^{11}\, \upmu$m$^3$/s (7 ml/min) in renal artery to $4 \times 10^{6}\, \upmu$m$^3$/s (4 nl/s) in afferent arterioles (AA). (**c**) Visualization of pressure distributed across the network and (**d**) its distribution (at the outlet of each vessel) vs Strahler order, ensuring smooth pressure drop from 100 mmHg at the inlet to a minimum of 30 mmHg at the end of afferent arterioles (AA). In the left panels (**a**,**c**), each vessel is visualized by a separate cylinder with a thickness corresponding to its radius, and color coded by the flow (**a**) or pressure (**c**), visualized by ParaView^28^.

To examine the physiological properties of the generated tree, we plot the blood flow and pressure distribution in Fig. 3. The flow associated with each vessel is derived from the zero-addition rule (cf. Eq. (8)) and the assumption of equal flow distribution among the afferent arterioles. As shown in Fig. 3a, the blood flow over our generated renal arterial network ranges from $1.2 \times 10^{11}\, \upmu$m$^3$/s (7 ml/min) in the renal artery to around $4 \times 10^{6}\, \upmu$m$^3$/s (240 nl/min) in afferent arterioles (AA).

We further plot the flow in each vessel vs the Strahler order in Fig. 3b (in log scale), which shows a clear exponential increase in flow with Strahler order. Although we have found no literature on such statistics in the rat kidney, this exponential increase is in close agreement with^35^ which measures the coronary blood flow vs Strahler order.

The pressure drop $\Delta p_i$ along each vessel *i* in the generated vascular tree can be computed by Hagen-Poiseuille’s law, cf. Eq. (5). Therefore, given the boundary condition of the inlet pressure $p_0$, the pressure value at every node along the generated tree can be computed by a simple breadth-first-search with $p_{i+1} = p_i - \Delta p_i$, where $p_{i+1}$ and $p_{i}$ denote the pressure at the outlet and inlet of vessel *i* respectively. From the literature^29^, pressure in the renal artery is around 90–110 mmHg, we hereby assumed an inlet pressure $p_0 = 100$ mmHg.

The pressure at each node in Fig. 3c shows a smooth decrease from 100 mmHg to a minimum of around 30 mmHg along the network without abrupt changes, indicating that the reconstructed vascular network produces physiologically feasible hemodynamic behaviors.

We plot node pressure (in mmHg) at the outlet of each vessel vs Strahler order of the generated renal arterial network in Fig. 3d, which shows a smooth and linear increase from around 55 mmHg at the end of afferent arterioles (Strahler order 0) to near 100 mmHg at the end of vessels with Strahler order 9, which then becomes flatter at the last order. This is expected because the root vessel has a short length *l* and a large radius *r*, resulting in a small pressure drop.

**Figure 4:**
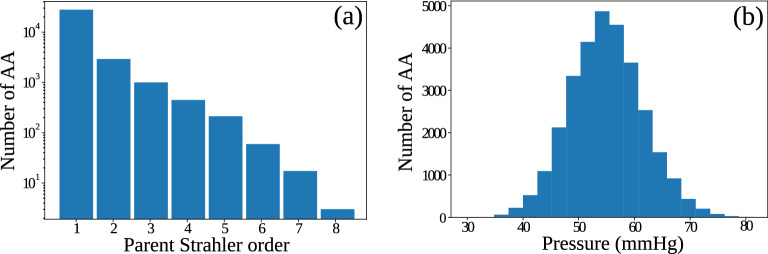
Morphometric and physiological features of afferent arterioles (AA) in the generated renal vascular network. (**a**) Number of afferent arterioles (in log scale) branching from the parent vessel of a given Strahler order in the generated tree. (**b**) Histogram of the pressure distribution among afferent arterioles.

We further plot the histogram of the pressures at the end of the afferent arterioles in Fig. 4b. Experimental data^29^ shows that the pressure at the end of the afferent arteriole is around 50–55 mmHg, which is in close agreement with the mean value from our result. However, since we only have a reasonable topological structure of the vascular tree but have not modeled the active regulation of pressure in the vascular tree , the histogram shows a wider distribution than found experimentally. In the future, such regulation and interaction among contiguous afferent arterioles^13,16^ need to be modeled to include the fine-tuning of the pressures and radii of the afferent arterioles.

## Discussion

We propose a hybrid framework for the reconstruction of the arterial vascular network of a rat kidney. The framework generates a full-scale 3-D vascular tree model based on a modified Global Constructive Optimization algorithm while taking image-based priors from a subject-specific scan. The hybrid method preserves subject-specific information by taking both the kidney’s shape and the main artery segmentation from micro-CT images of a real rat into the initialization step.

The reconstructed vascular tree shows good morphometric agreement with anatomical data from a real rat kidney^7^. Furthermore, the calculated pressure distribution throughout the vascular tree is in good agreement with values found experimentally^29^. Whereas the overall topology is in good agreement with the experimental observations, there are some deviations between the values, especially the vessel radii. In contrast to the topology of the vascular tree, vessel radius is a dynamic variable that is determined by the local conditions in the tissue. Thus, vessel radius may vary considerably from time to time in a given vessel, and the measurement process itself, e.g., the injection of casting material, may in itself cause changes in the radius. Nonetheless, the largest deviation was in the root vessel, which does not play a significant role in renal hemodynamics. Its resistance is negligible compared to the smaller vessels in the tree and, therefore, of little significance for total renal blood flow.

It could be argued that vessel radius should be part of the cost function, and thus be optimized in the construction of the tree. However, we found that incorporating the radius in the cost function defined in Eq. (7), in fact, deteriorated the outcome of the optimization process, see “Physiologically based cost functions”. One reason could be that the cost function is local, whereas the flow is determined not only by local factors but also by the global physiological demands on the organ. This suggests that an optimal procedure could be to construct a realistic vascular tree using an algorithm like the one proposed in the present paper, followed by an optimization of the vessel radii based on a global physiological target. In the kidney, this could be the resulting glomerular filtration rate or a similar global measure of renal function.

Importantly, the reconstructed structure is not a simple bifurcating tree, a structure which previously has been shown to be insufficient for supplying blood to the glomeruli at a sufficient pressure^13^. Instead, in agreement with previous work on the structure of the renal vascular tree^1^, afferent arterioles arise not only from terminal arteries (Strahler order 1), but also from all arteries of higher order, except for the largest arteries (Stahler order 9 and 10) (cf. Fig. 4a). Therefore, the proposed method can generate both morphometrically correct and physiologically feasible vascular trees while respecting the prior information from the subject scan.

Modifications of subprocesses can easily be integrated into our framework. For example, although the current image prior requires a semi-automated segmentation of the main arteries, one can replace it with state-of-the-art deep learning models, e.g., UNet^36^ to do the auto segmentation if one has accurate training data. Similarly, if renal cortex segmentation is feasible in a different scanning setting, one could skip the cortex extraction (Eq. 10) step and directly do the sampling of terminal nodes over the segmented cortex.

Future work will be to apply the reconstructed network in the areas described in the previous section, e.g., to model the active regulation of pressure and flow in our generated arterial tree^13,16^. We have focused on the rat kidney since it is the only one for which detailed data on the vascular tree are available for validations^7,29^. Nonetheless, we expect that our model can generalize to the human kidney as well, given similar micro-CT scans with similar resolution. The renal medulla imposes intrinsic constraints on artery growth, which must be addressed in the CCO or GCO process. However, since our network starts with a pre-built tree, the vessels will never pass these regions as long as the vessel segmentation is accurate to a certain extent, giving piece-wise convex regions in the initialization process.

With a full-scale tree structure, it is the ultimate goal to model pathological changes in the kidney. For example, at the structural level, it could be reducing the number of terminal vessels to mimic loss of nephrons, or modifying the radii of certain vessels while simulating the resulting changes of pressures and flow to mimic renal artery stenosis and atherosclerotic changes in the kidney. However, the pathological changes may be functional, e.g., a reduced glomerular filtration or tubular reabsorption rate. To include these types of pathology, it is necessary to expand the model with models of the nephrons. This will be part of the next step because, in addition to the structure of the vascular network, it will require an additional model of functioning nephrons attached to each afferent arteriole together with the relevant regulatory systems, e.g., the myogenic mechanism and tubuloglomerular feedback^37^.

Another future direction is to create a synthetic renal vessel dataset by generating the ground truth segmentation labels corresponding to the generated tree. To create such an image dataset, we need to remap the reconstructed vascular tree back to a smooth surface mesh or binary label map. The details of such a process and an example of an image-label pair (Fig. S2) are given in the Supplementary material. We will test whether these artificially generated vessel images can be used to pre-train a deep-learning-based segmentation network for transfer learning or to train a Generative Adversarial Network (GAN) for domain transfer. As an example, Menten et al.^2^ recently applied CCO to synthesize retinal vascular plexuses and generated corresponding Optical Coherence Tomography Angiography (OCTA) images by emulating the OCTA acquisition process. They showed that these simulated data can successfully pre-train a retinal vessel segmentation network to segment real OCTA retinal images.

**Figure 5:**
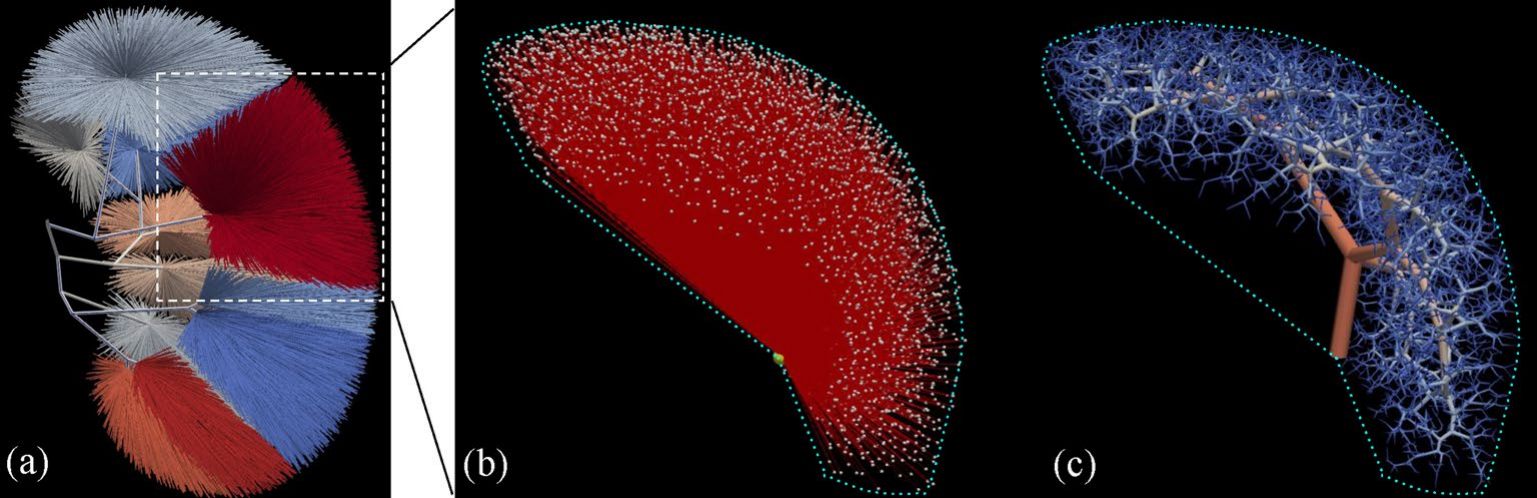
Illustration of the initialization and result of a subtree, visualized by ParaView^28^. (**a**) The initialization of the whole tree from Fig. 1h. (**b**) The initialization of one subtree from (**a**), zoomed in and rotated. (**c**) The result of the subtree, which can only grow inside the outlined region defined in (**b**), thus it will satisfy the convex constraint, and will not penetrate the renal medulla or grow outside the kidney structure.

Upon finishing our work, we also notice that each subtree inside each piece-wise convex region after the initialization step of our GCO method (cf. each colored subtree in Fig. 1h) is independent of each other. Specifically, the leaf nodes that are connected to a certain node of the pre-built tree in the initialization step will always belong to the successors of that node. Figure 5 shows an example of the initialization and the result of one subtree. Here, the resulting subtree will stay inside the region defined in the initialization step with the same group of leaves and is independent of other subtrees. Although they all belong to a larger tree with a single root vessel, each subtree can be optimized independently in parallel before being merged together in the end. This parallelization has not been implemented explicitly, which should also be a future direction to speed up the whole computation.

The subtrees created by the initialization process are reminiscent of the vascular dominant regions found in the kidney^38,39^. When planning surgical removal of part of the kidney, surgeons identify the first few arterial branches from the renal artery to estimate the subregion supplied by each of the branches^38,39^. Each subregion is assumed to only get blood supply from the closest branch. These independently supplied regions resemble the piece-wise convex regions in our initialization step for the growth of the subtrees (Fig. 1h). Similar to our pre-processed arterial tree (Fig. 1g), these procedures only identify the first few (around 3) branches from the renal artery^38,39^. Otherwise, the vascular dominant regions become less well-defined.

The pair-wise coupling of the arterial and venous systems is not trivial to integrate, when in the future extending the framework to cover both arteries and veins. Currently, it is only possible to independently generate two individual trees. This does not capture the pair-wise coupling of arteries and veins, nor does it avoid the early intersection of the two trees. Kretowski et al. applied a CCO-based approach to create complementary hepatic arterial and venous trees^40^. They detect and avoid intersections between the two trees explicitly during the growing process by adjusting the radius of each vessel at the expense of violating Murray’s law (Eq. (9)). This process is time-consuming and needs modification over the GCO process, where vessels are optimized on a larger scale.

Finally, we can generate more realistic trees by utilizing more vessels from the segmentation of the scans, or by having a better estimate of the renal cortex region in the initialization step. Currently, we are deliberately removing a large portion of the extracted centerline to only preserve the main arteries, since the other parts are prone to noise and difficult to detect by later auto-segmentation methods. If we have better segmentations, these small segments could also be used to guide the reconstruction of subject-specific vascular networks. On the other hand, one could also experiment with how small a portion of a pre-built tree we need in the initialization step of GCO before it will violate anatomical constraints, e.g., pass through the renal pyramid or outside the kidney structure without the piece-wise convex premise. Another potential future direction involves integrating large vessels in a totally different manner. Instead of a pre-built tree, the segmentation could also be incorporated in the cost function as gravitation to guide the whole process by “pulling” the intermediate nodes close to their positions. However, it is non-trivial to add such pull-force to Eq. (7) while balancing the other constraints. It will probably involve lots of testing of the parameters to find such a balance.

## Methods

The original CCO algorithm works by iteratively adding a new edge (vascular segment). After each addition, the newly created bifurcation is locally remodeled and all tree radii are adjusted geometrically. Such remodeling is clearly inefficient when the vessel structure becomes large like in the kidney. In contrast, we adopt the alternative GCO algorithm as our backbone model. This method overcomes the problems of the CCO by starting with a fully connected tree, where the leaves are usually either defined on a regular grid or randomly positioned within the organ hull^6^. In our case, the leaves are sampled using Poisson disk sampling^26^ from the estimated renal cortex (Fig. 1c) as detailed in “The leaf node sampling”. It performs a multi-scale optimization to find an optimal tree for all leaf nodes simultaneously and introduces a global pruning operation after each iteration to produce a new tree with better global branching structures.

In this section, we start by stating the assumptions and objectives of the constructive algorithm (GCO). We then explain the underlying cost function to be optimized by GCO and the general process of GCO, including the modifications we have made. We then present in detail how the image priors are integrated into our hybrid framework and end by introducing the utilized scan information and implementation details.

We do acknowledge that there are several approximations involved in the whole process, e.g., all the assumptions defined in “Assumptions and objectives”, the approximation algorithm involved in the splitting process in “Global Constructive Optimization algorithm”, as well as the cortex approximation in “The image priors”. We will explain the underlying rationale or why they are inevitable when introducing these approximations.

### Assumptions and objectives

Several assumptions have to be made for the whole process of reconstructing vascular trees. The first assumption forms the basis of the mathematical modeling of any vascular tree and has been adopted in most of the angiogenesis-based methods, e.g., on liver, heart, and brain^4,6,23,24^. The second assumption comes from structural and functional properties of the kidney that glomeruli and afferent arterioles of all nephrons are located in the cortex region^41,42^. It is the rationale for a Poisson disk sampling of terminal nodes (glomerulus), which will be discussed in “The leaf node sampling”. The other assumptions relate to the hemodynamics and are necessary to satisfy Poiseuille’s equation in Eq. (5), which is an integral part of the cost function Eq. (7).

- A vascular tree is modeled as a collection of connected, straight cylindrical tubes, indicating constant radius and no curvature between branch points.
- All the renal arteries end in the renal cortex with a certain perfusion territory.
- Blood is incompressible and Newtonian, and blood flow is laminar.
- Pressure drop due to branching is negligible.
- Flows are equally distributed among each terminal vessel.

Given such assumptions, a vascular tree is modeled by a directed acyclic graph ${\mathcal {G}} \equiv ({\mathcal {V}}, {\mathcal {E}})$ where ${\mathcal {V}}$ is a set of nodes at the endpoints of each vessel centerline with node features being its coordinates in Euclidean space, and ${\mathcal {E}}$ is a set of directed edges which form a connected tree structure. Each edge represents a single vessel segment as a cylinder with its radius and flow as the edge feature. Note that length is not modeled as an edge feature but rather derived from the Euclidean distance between the two end nodes of each edge. The goal is to find a tree that minimizes the system’s overall cost function while fulfilling the constraints. Specifically, given the position of a single root node *s*, and *n* leaf nodes $l_i$, the goal is to find a tree ${\mathcal {G}} \equiv ({\mathcal {V}}, {\mathcal {E}})$ that contains *s* and $l_i \in {\mathcal {V}}$ with minimum cost defined in the next subsection and fulfills constraints by introducing new intermediate nodes $v_i \notin \{s, l_1, \cdots , l_n\}$ and connections (edges) ${\mathcal {E}}$.

In our work, we propose a novel way to integrate image priors into the initialization of GCO, so that the input is no longer a single root with *n* leaves, but a pre-built tree ${\mathcal {G}}_0 \equiv ({\mathcal {V}}_0,{\mathcal {E}}_0)$ with $s \in {\mathcal {V}}_0$ that already covers the main arteries.

### Physiologically based cost functions

Our reconstruction method generates subject-specific arterial vascular networks ${\mathcal {G}} \equiv ({\mathcal {V}}, {\mathcal {E}})$ under the optimality assumption that the network structures will maintain adequate blood perfusion with minimal total expense along all its edges, which can be approximated by the total sum of the local cost at each branching node *v*:

1 $$\begin{aligned} C({\mathcal {G}}) \equiv \sum _{v} C_{\text {local}}(v) \equiv \sum _{v} \sum _{e \in {\mathcal {B}}_{v}} C(e) \, \end{aligned}$$

where ${\mathcal {B}}_{v}$ denotes the set of all the incident edges of node *v*.

**Figure 6:**
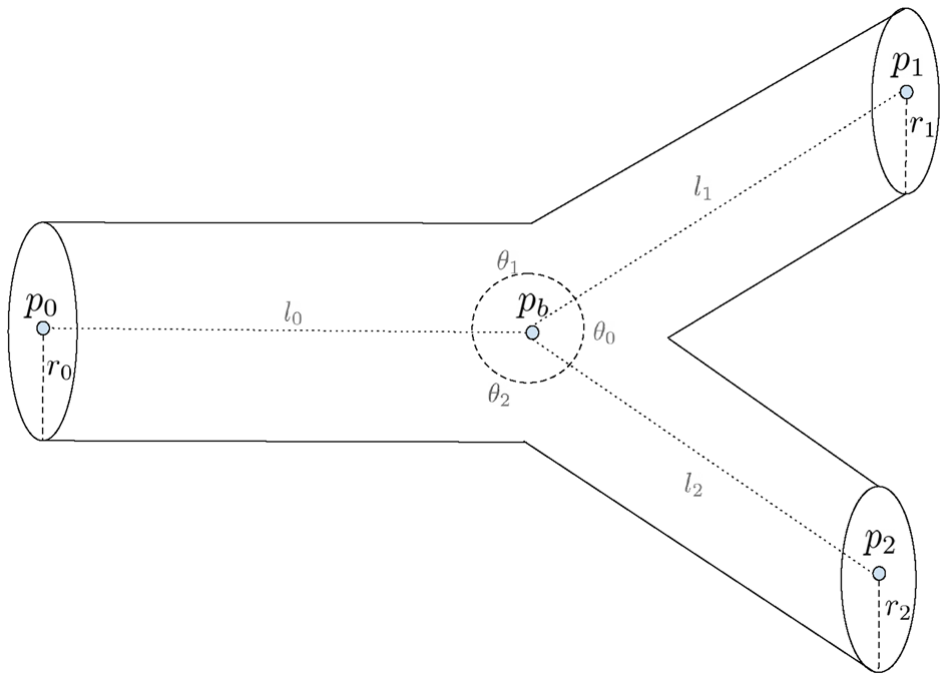
A typical vessel branching model. The branching vessels are uniquely defined by the locations of the three end nodes ($p_0, p_1, p_2$), the location of the bifurcation node ($p_b$), and the radii of the three incident edges ($r_0, r_1, r_2$). Length ($l_s$) and branching angles ($\theta _s$) are not modeled explicitly but can be trivially derived.

A typical branching model is shown in Fig. 6. Note that the GCO model does not enforce bifurcation explicitly and can indeed model any number of branches $>2$. Still, branches other than bifurcations or trifurcations are rarely seen in the final result, because they usually incur a higher cost. In each branching model, an optimal branching point is positioned with respect to fixed neighboring edge radii and neighboring node positions to minimize the cost function. Following the work of Tekin and Shen et al.^24,32^, we incorporate both the material cost ($M_{loss}$) and power cost ($P_{loss}$), resembling the biological infrastructure cost to build the vessel and the power dissipated during blood circulation, respectively. Therefore, $C_{\text{ local }}(v)$ is a weighted combination of the two costs:

2 $$\begin{aligned} C_{\text{ local }}(v) \equiv w_c \, M_{loss}(v) + w_p \, P_{loss}(v) \end{aligned}$$

where $w_c$ and $w_p$ are the weight factors to balance the two costs. $M_{loss}(v)$, which expresses the amount of materials that constitute the blood in the vessels, is only dependent on the intravascular volume of the arterial tree. It is given by

3 $$\begin{aligned} \begin{aligned} M_{loss}(v) \equiv \sum _{e \in {\mathcal {B}}_{v}} M(e) \equiv \sum _{e \in {\mathcal {B}}_{v}} \pi r_e^2 l_e \, {.} \end{aligned} \end{aligned}$$

Analogous to electric power, the power dissipated during blood circulation ($P_{loss}(v)$) is defined by the product of flow $Q_{e}$ (analog to electric current) and pressure drop $\Delta p_e$ (analog to potential difference),

4 $$\begin{aligned} P_{loss}(v) \equiv \sum _{e \in {\mathcal {B}}_{v}} Q_{e} \Delta p_e \end{aligned}$$

where Hagen-Poiseuille’s law gives the pressure drop $\Delta p_e$ necessary to overcome the resistance to flow due to the viscosity$\mu$, of the blood in an individual blood vessel,

5 $$\begin{aligned} \Delta p_e = \frac{8 \mu l_e Q_e}{\pi r_e^4} \, {.} \end{aligned}$$

Combining Eqs. (4) and (5) gives

6 $$\begin{aligned} \begin{aligned} P_{loss}(v) \equiv \sum _{e \in {\mathcal {B}}_{v}} Q_e^2 \frac{8 \mu l_{e}}{\pi r_{e}^{4}} \, {.} \end{aligned} \end{aligned}$$

The total cost at each branching *v* is a weighted combination of material cost Eq. (3) and power cost Eq. (6),

7 $$\begin{aligned} \begin{aligned} C_{\text{ local }}(v)&\equiv w_c \, M_{loss}(v) + w_p \, P_{loss}(v) \, \\&= w_c \, \left( \sum _{e \in {\mathcal {B}}_{vx}} \pi r_e^2 l_e \right) + w_p \, \left( \sum _{e \in {\mathcal {B}}_{v}} Q_e^2 \frac{8 \mu l_{e}}{\pi r_{e}^{4}} \right) \, \\&= \sum _{e \in {\mathcal {B}}_{v}} \left( w_c \pi r_e^2 l_e + w_p Q_e^2 \frac{8 \mu l_{e}}{\pi r_{e}^{4}} \right) . \end{aligned} \end{aligned}$$

Blood flow follows the simple zero-addition rule from Kirchhoff’s first law, from which we derive the relation,

8 $$\begin{aligned} Q_p = \sum _{e \in {\mathcal {B}}_{v}/p} Q_e \end{aligned}$$

where *p* denotes the parent edge in ${\mathcal {B}}_{v}$ of node *v*. Note that radii are not optimized but assumed to follow Murray’s law at the branch points^5,20^,

9 $$\begin{aligned} r_p^3 = \sum _{e \in {\mathcal {B}}_{x}/p} r_e^3 \, {.} \end{aligned}$$

Although we recognize that Murray’s law is only an approximation, we found that optimizing radii, e.g., by integrating an equality constraint into the optimization process as proposed by^24^ deteriorates the result by producing abrupt changes in the radii at branch points, and resulting in a final tree with fewer than the 11 Strahler orders found experimentally^7^.

In our experiment, we set the weight factors $w_c=5 \times 10^{4}\, \mathrm {~J} \mathrm {~s}^{-1} \mathrm {~m}^{-3} = 5 \times 10^{-8}\, N \, \upmu$m$^{-2}$ s$^{-1}$, and $w_p=1$, as we have found that they result in the same scale of the two cost terms. We also adopt constant viscosity $\mu =3.6 \times 10^{-3}\, \mathrm {~Pa} \, \mathrm {~s} = 3.6 \times 10^{-15} \,\mathrm {N \, s} \, \upmu$m$^{-2}$, and inlet flow $Q_0 =$ 7ml/min = $1.167 \times 10^{11}\, \upmu$m$^3$   s$^{-1}$ with values from the literature^43,44^. Assuming equal flow distribution over terminal vessels, flow at afferent arterioles can be calculated as $Q_t = \frac{Q_0}{N}$ where *N* is the number of afferent arterioles. In our case, $Q_t =\frac{1.167 \times 10^{11}\, \upmu \text {m}^3 \,\text { s}^{-1}}{3 \times 10^4}= 3.89 \times 10^6\, \upmu$m$^3$   s$^{-1} = 3.89$ nl   s$^{-1}$, complying with the literature where $Q_t \approx \ 4$ nl s$^{-1}$^45^. Note that the physics units and voxel size need to be consistent with each other to ensure that $M_{loss}$ and $P_{loss}$ are on the same scale. The above units give both $M_{loss}$ and $P_{loss}$ in the scale of $N \, \upmu$m   s$^{-1}$ ($\mu W$).

The combination of the two losses is in correspondence with the derivation of Murray’s law^5,20^, and is based on a compromise between the power required to drive flow through the vessel ($P_{loss}$) and the rate of expenditure of metabolic energy required to maintain the volume of blood filling the vessel ($M_{loss}$). Although the original CCO and GCO methods only consider $M_{loss}$, $P_{loss}$ is vital for a vascular system that requires efficient flow^32^ such as in the kidney. Experimentally we have also found it necessary to integrate $P_{loss}$ into the loss function, since using $M_{loss}$ alone (or a too large weight for $M_{loss}$ ) does not generate anatomically correct tree topologies, e.g., giving only 9 Strahler orders instead of 11 as reported in^7^. Note that the blood supply cost, defined in^46^ to produce evenly dispersed terminal nodes, is not needed in our model, since the terminal nodes have already been sampled by Poisson disk sampling^26^, which maintains a minimum distance among them, as discussed in “The leaf node sampling”.

### Strahler ordering

The Strahler ordering method is a common method for labeling trees with a hierarchical structure, e.g., a vascular tree^7,8,47^. It begins at the top of the tree by labeling all the leaves (the afferent arterioles in this case) as having Strahler order 0. A 0 order vessel has no vessels branching from it. Following the tree upstream, the order of the parent vessel (edge) increases by one order to $j+1$, if two or more of the daughter vessels are of order *j*, where *j* is the highest order among the daughter vessels. Otherwise, the parent vessel takes the highest order (*j*) among its daughter vessels. The process is continued until it reaches the root of the tree. Note that this ordering method is not used in the reconstruction algorithm itself, but is used for the quantitative comparison of the reconstructed tree with the measurements of a renal vascular tree reported in^7^.

### Global Constructive Optimization algorithm

The Global Constructive Optimization (GCO) algorithm includes the following steps, which are iterated multiple times before convergence except for the first initialization step. Please refer to^6^ for a more detailed explanation of the process.

1. *Initialization.* In the original GCO initialization, each sampled terminal node is connected to a single user-defined root node, thus completely ignoring subject-specific information. In our hybrid framework, the sampled terminal nodes are connected to the main arteries derived from the patient’s scan. Details of the sampling process and main arteries retrieval are explained in “The image priors”.
2. *Relaxation.* The relaxation process finds the best location for each branching node through optimization by minimizing the overall cost function defined in “Physiologically based cost functions”.
3. *Merging.* Merging involves contracting the edge between two neighboring nodes. It is applied when the ratio between the shortest incident edge of a node and the second incident edge is within a threshold, which usually happens when relaxation places a node at the same location as one of its neighboring nodes.
4. *Splitting.* Splitting is done whenever creating a new intermediate node and reconnecting a subset of the original child neighbors $S \in {\mathcal {B}}_v/{p}$ introduces a lower cost. Usually, this condition is fulfilled at a node with too many edges, indicating that bifurcation is implicitly imposed on the modeling. However, finding the optimal subset is in ${\mathcal {O}}(n!)$ thus NP-hard. Instead, an approximation algorithm is applied by first finding a subset $S_1 \in S$ with two edges that introduce the lowest cost. A new edge is then iteratively added to $S_n$ from $S-S_{n-1}$ if it introduces a lower cost. This approximation has a complexity of ${\mathcal {O}}(n^2)$ thus much more efficient. It is worth mentioning that this operation is still more computationally heavy than the actual optimization step in relaxation.
5. *Pruning.* A pruned tree ${\mathcal {G}}_l \equiv ({\mathcal {V}}_l, {\mathcal {E}}_l)$ is created from ${\mathcal {G}}$ by removing all edges deeper than some threshold. This process will only keep the large branches generated from each iteration and produce a new tree with a better global branching structure in the next iteration. Here we start by keeping the first two branches of each subtree while keeping one more branch after every two iterations. All the leaf nodes that are removed in this operation are reconnected to the nearest node in the pruned tree. The modification we make here is that each leaf node can only be reconnected to the subtree that it belongs to in the initialization step.

### The image priors

In general, our proposed hybrid way of utilizing image-based priors involves two segmentation maps from the kidney scans, to begin with, as shown in Fig. 1: segmentation of large arteries $Y_a$ (Fig. 1e), and segmentation of the whole kidney structure $Y_w$ (Fig. 1a). The segmentation of large arteries is obtained using a semi-automated approach^48^. Whole kidney structure segmentation, however, is obtained by simple thresholding, since the ex-vivo micro-CT scan makes it easy to separate the kidney from the background. These two segmentation maps are used in the following two tasks for the initialization of the whole GCO process. Figure S1 in Supplementary shows the flowchart of the process.

#### The leaf node sampling

The segmentation of the whole kidney structure $Y_w$ is used to sample terminal nodes where the arteries end (Fig. 1d). Since the arteries end in the cortex region rather than only on the surface, to mimic the anatomical rules, several more steps are necessary.

##### Cortex approximation via erosion 

The renal arteries end in the renal cortex, which requires a cortex segmentation to sample from. Since the cortex is not visible from our micro-CT scan, it is approximated by a certain distance ($R_1 \approx 2 \ m m$) away from the surface by assuming equal thickness across the kidney. The thickness of the rat kidney cortex depends on the age, sex, and size of the rat. A value of 2 mm is typical for a 12–18 week-old rat^49,50^. This is the age group typically used experimentally. This process can be easily obtained by the subtraction of a mathematical erosion ($\cdot$) applied to $Y_w$, as shown in the yellow regions in Fig. 1b.

##### Inner region removal

 To avoid sampling terminal artery nodes around the renal artery, all the regions near a certain distance to the root node $v_r$ are removed. This is accomplished by imposing a ball centered at $v_r$ ($R_2 \approx 5.65 \ mm$), as shown in the green regions in Fig. 1b. The size of the region is based on our scans of the kidney and provides an automatic means to avoid sampling afferent arterioles near the hilus. Specifically, $R_2$ has to be large enough to cover the hilar region that belongs to the segmentation $Y_w$ but is not part of the cortex. We note that it also removes parts of the kidney cortex near the hilus. Despite this, we find it an appropriate approximation for the cortex geometry, especially as the results are not critically dependent on this parameter. In summary, cortex segmentation is a set of points

10 $$\begin{aligned} Y_c \equiv \left\{ ~{\textbf{x}}~ \mid ~ {\textbf{x}} \in {Y_w} - ({Y_w} \cdot R_1)~\wedge ~ \Vert {\textbf{x}} - {\textbf{v}}_r\Vert _2 > R_2 \right\} \end{aligned}$$

of which the surface mesh is visualized in Fig. 1c.

##### The Poisson disk sampling

 Vessels inside an organ follow an anatomical structure that the leaf nodes should cover the entire perfusion territory while avoiding being too close to each other to prevent competition or overlap between branches^46^. This is important as one of the main purposes of the vascular tree is to supply blood to all the tissues of an organ^46^. With a fixed perfusion territory, i.e., the extracted cortex $Y_c$ from the previous step and a fixed number of terminal nodes, evenly spreading the terminal nodes inside the cortex volume is the most straightforward way to mimic this anatomical property. Poisson disk sampling^26^ maintains a minimum distance between sampled points by sampling from the spherical annulus of existing points and rejecting points that are too close to each other, which resembles such an anatomical rule very much. In the present model, the minimum distance value can be approximated from the cortex volume and the number of points we would like to sample from. We follow the work of Nordesletten^7^ by sampling 30K terminal vessels (number of arteries with Strahler order 0), which results in a minimum distance of around 270 $\upmu$m. Note that this is the distance between the distal end of afferent arterioles, and not the gap between the glomeruli that originates from the terminal nodes. Monte Carlo sampling is also adopted to do Poisson disk sampling over the whole cubic volume before filtering out the points from non-cortex regions.

#### Large artery extraction

To integrate the large artery segmentation $Y_a$ into the GCO process, vessels need to be modeled by a graph with nodes along the centerline and edges with connectivity information. Therefore, we start by extracting the centerline from $Y_a$ using the Skeletonization method proposed by Bærentzen et al.^51^. Instead of a binary image with width 1 at each local foreground voxel, the algorithm outputs a graph data structure $C(Y_a) = {\mathcal {G}}({\mathcal {V}}, {\mathcal {E}})$ suitable for our needs. However, extracting a tree structure via skeletonization of segmentations is difficult. Intense manual work must be involved afterward, even with accurate segmentation label maps. Therefore, when we have very coarse segmentation $Y_a$ without clean resolution, only the first few branches from the root are trustworthy, while veins can be falsely segmented as arteries in the deeper branches. Moreover, the extracted centerline (Fig. 1f) is an undirected graph and may contain loops, which cannot be directly used. Several preprocessing operations are necessary before initialization.

##### Minimum spanning tree

 This first operation removes potential loops by creating a subset of the edges with the minimum total edge weight from ${\mathcal {G}}({\mathcal {V}}, {\mathcal {E}})$ that connects all the vertices without any cycles. Here the weight of each edge is the negative of its radius derived from a Euclidean distance transform over the derived centerline to the segmentation. This operation will remove the thinnest edge to break any loop. After this step, the acyclic graph can be converted to a tree (directed acyclic graph) with a simple depth-first-search.

##### Intermediate nodes removal

 As stated in the assumption from “Assumptions and objectives” that each vessel is modeled by a straight cylindrical tube, any intermediate nodes along each single vessel will have to be removed. This will, of course, introduce artifacts to the length computation, but is assumed to be negligible within a reasonable curvature.

##### Degree pruning

 For each node with more than 4 branches, we only keep a maximum of 4 longest paths, since branching into more than 4 children is not realistic. Specifically, Marsh et al.^9^ found at most 4 branchings, while Nordsletten et al.^7^ modeled up to trifurcations.

##### Depth pruning

 For each node, we compute its cumulative distance to the root along the tree and only keep nodes up to a certain distance, which we set around 10 K $\upmu$m (450 voxels with a $22.6 \upmu$m voxel size). Such threshold is experimentally determined to keep only the first few branches which are noise-free. The rationale is that even though some thin vessels far away from the root are visible from the current segmentation, only large vessel segmentation is trustworthy. Especially if we would like to further adapt deep learning for automatic vessel segmentation, we cannot assume the model to be able to detail the thin vessels.

##### Connected component decomposition

 The two pruning operations may introduce smaller disconnected trees, we thus only keep the largest tree.

#### Final GCO initialization

For the initialization of GCO (Fig. 1h), all the sampled terminal nodes (Fig. 1d) are connected to the nearest ending node along the extracted and pre-processed large artery centerline graph $\mathcal {G'}({\mathcal {V}}, {\mathcal {E}})$ (Fig. 1g). The radii associated with the terminal vessels are sampled from a Gaussian distribution $r_0 \sim {\mathcal {N}}(10.08, 0.14)$ derived from literature^7^, while radii of other vessels are derived from the radii of terminal vessels by Murray’s law (Eq. (9)).

Besides retaining subject-specific information from image priors, the connection to the pre-built tree also makes the complex structure piece-wise convex, making the later constructive algorithms applicable here. Specifically, the connection between any terminal node to the pre-built tree should not enter or cross the renal pyramid, which is hard to satisfy when the pre-built tree is only a single root node.

The pruning operations in the previous subsection remove a large portion of the deep branches, only preserving the main arteries. This is necessary due to the noisy input. Nonetheless, the remaining large branches are enough to satisfy the piece-wise convex constraint in the initialization step. Specifically, the connections between the sampled terminal nodes to the nearest node in the large artery centerline graph $\mathcal {G'}({\mathcal {V}}, {\mathcal {E}})$ naturally avoid passing through the renal pyramid or going outside the kidney structure, as shown in Fig. 5. The GCO process afterward will remain in each convex shape created in the initialization step because moving outside will always enforce a larger cost as defined in “Physiologically based cost functions”.

### Ex vivo micro-CT imaging dataset

The kidney cast was prepared as described in^48^ in agreement with approved protocols (approval granted from the Danish Animal Experiments Inspectorate under the Ministry of Environment and Food, Denmark). The rat kidney was ex vivo scanned in a ZEISS XRadia 410 Versa $\upmu$CT scanner (Carl Zeiss Microscopy GmbH, Jena, Germany) at the following settings: isotropic voxel size 22.6 $\upmu$m, 50 kV tube voltage, 0.2 mA current, appertaining LE3 filter, 360$^{\circ }$ scan around the vertical axis with 3201 different projections (0.112$^{\circ }$ rotation steps)^48^. The raw scan has a dimension of $1000 \times 1024 \times 1014$ voxels. To ease the computational overhead, the scan is auto-cropped to $955 \times 508 \times 626$ by an intersected bounding cube of the largest component from simple Otsu’s thresholding over maximum intensity projections to three dimensions.

### Implementation details

Shen et al.^24^ and Keelan et al.^46^ proposed to optimize the cost function using Simulated Annealing, which is a metaheuristic to approximate the global optimum of a given function. Because of its non-gradient-based nature, this method is usually preferable for problems where gradients are hard to compute. However, we note that the cost function defined in Eq. (7) is quite differentiable, meaning its gradient can be easily computed analytically, giving

11 $$\begin{aligned} \begin{aligned} \nabla C_{\text{ local }}(v)&= \nabla \left( \sum _{e \in {\mathcal {B}}_{v}} \left( w_c \pi r_e^2 l_e + w_p Q_e^2 \frac{8 \mu l_{e}}{\pi r_{e}^{4}} \right) \right) \, \\&= \sum _{e \in {\mathcal {B}}_{v}} \left( w_c \pi r_e^2 + w_p Q_e^2 \frac{8 \mu }{\pi r_{e}^{4}} \right) \nabla \, \Vert {\textbf{v}} - {\textbf{n}}_{v, e}\Vert _2 \, \\&= \sum _{e \in {\mathcal {B}}_{v}} \left( w_c \pi r_e^2 + w_p Q_e^2 \frac{8 \mu }{\pi r_{e}^{4}} \right) \frac{ {\textbf{v}} - {\textbf{n}}_{v, e} }{ \Vert {\textbf{v}} - {\textbf{n}}_{v, e}\Vert _2 } \end{aligned} \end{aligned}$$

where ${\textbf{n}}_{v, e}$ denotes the position in ${\mathcal {R}}^3$ of the neighboring node of *v* along edge *e*. Therefore, we apply the standard Broyden–Fletcher–Goldfarb–Shanno (BFGS) method which proves to perform as well and is much faster. Figure S3 in Supplementary shows the convergence plot of the GCO process.

All the backbones are pure NumPy and SciPy-based computation, with graph representation using NetworkX^52^. The computations were conducted in Ubuntu 22.04 with an Intel Core i7-8700 processor at 3.20 GHz and 24 GB RAM. Currently, there is no GPU acceleration. In fact, the optimization process in relaxation is not the bottleneck. As discussed in “Global Constructive Optimization algorithm”, splitting is usually the computational bottleneck and dominates the time complexity, especially in the first few iterations where there are a small number of intermediate nodes each with a large number of neighbors. Moreover, since each branching has to be optimized individually and consecutively, switching to PyTorch with GPU acceleration will not help. The process of integrating image priors, such as centerline extraction and Poisson disk sampling, takes approximately 1 h, while the GCO process after initialization takes approximately 10 h to reach convergence.

## Ethics approval

The experiments were conducted in agreement with approved protocols (approval granted from the Danish Animal Experiments Inspectorate under the Ministry of Environment and Food, Denmark). All procedures agreed with the ethical standard of the university, which meets that of the EU Directive 2010/63/EU for animal experiments.

## Supplementary Information

Supplementary Information.

## Data Availability

Raw data and image processing algorithms can be exchanged through a collaboration agreement to Carsten Gundlach (cagu@fysik.dtu.dk). Processed data and analysis algorithms can be made available upon request to Peidi Xu (peidi@di.ku.dk).

## Code availibility

The source code for the paper is available at https://github.com/diku-dk/RenalArterialRecon under Apache License 2.0 open-source license.
