# Supplementary material for: A hybrid approach to full-scale reconstruction of renal arterial network
Source: Sci Rep. 2023 May 9;13:7569. doi: 10.1038/s41598-023-34739-y (PMC10169837; doi:10.1038/s41598-023-34739-y)
Supplement: Supplementary file 1 — Supplementary Information. [file 41598_2023_34739_MOESM1_ESM.pdf]

Supplementary Material: A Hybrid Approach to Full-Scale Reconstruction of Renal Arterial Network

Peidi Xu[1*], Niels-Henrik von Holstein-Rathlou[2], Stinne Byrholdt Søgaard[2], Carsten Gundlach[3], Charlotte Mehlin Sørensen[2], Kenny Erleben[1], Olga Sosnovtseva[2†] and Sune Darkner[1†]

[1]Department of Computer Science, University of Copenhagen, Universitetsparken 1, Copenhagen, 2100, Denmark.
[2]Department of Biomedical Sciences, University of Copenhagen, Blegdamsvej 3B, Copenhagen, 2200, Denmark.
[3]Department of Physics, Technical University of Denmark, Kongens Lyngby, Copenhagen, 2800, Denmark.

*Corresponding author(s). E-mail(s): peidi@di.ku.dk;
Contributing authors: nhhr@sund.ku.dk; sbyrholdt@sund.ku.dk; cagu@fysik.dtu.dk; cmehlin@sund.ku.dk; kenny@di.ku.dk; olga@sund.ku.dk; darkner@di.ku.dk;
†These authors contributed equally to this work.

1 Visual animations

Visualizations of our generated renal arterial tree in 3D gif animations are available at https://github.com/KidneyAnonymous/RenalArterialTree. They are created by applying a Tube filter in ParaView [1] where each tube is assigned with the radius of the generated vessel segment. Different gifs represent the tubes colored by different properties, e.g., radius, flow, and pressure.

2 Flow chart

We show the flowchart of how the image priors are integrated into the GCO process in Fig. 1. The micro-CT scan is used to extract a prebuilt large arterial tree and sample leaf nodes from the two segmentation maps respectively with some intermediate steps. These two outputs are then the inputs to the GCO initialization step to guide the reconstruction of the full-scale arterial tree.

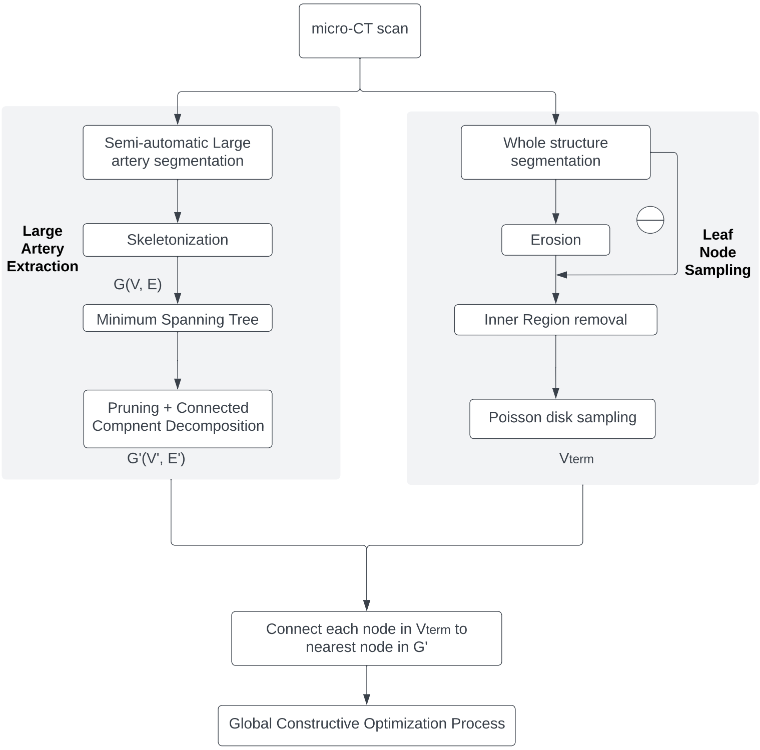

Fig. 1: Flowchart of the complete computational framework where ⊖ denotes element-wise subtraction. In the main text, the whole process is detailed in Section 4.5, while the final Global Constructive Optimization process is detailed in Section 4.5.

3 Artificial kidney vessel dataset

The generated vascular tree from our pipeline will vary between runs since there are several sources of randomnesses involved, e.g., the Poisson disk sampling of terminal vessels, its radii sampled from Gaussian distribution, and

the approximation algorithm in the splitting process. We can thus create a synthetic kidney vessel dataset by generating the ground truth segmentation labels corresponding to each generated tree. To create such an image dataset, we need an inverse task to remap the reconstructed vascular tree $\mathcal{G} = (\mathcal{V}, \mathcal{E})$ back to a smooth surface mesh or binary label map. This is usually much more complicated than simply stacking each individual cylinder together. A reconstructed vascular tree $\mathcal{G} = (\mathcal{V}, \mathcal{E})$ hosts the centerline location as well as the radius of its maximal inscribed spheres. The way we generate a smooth surface structure is by constructing a tube function based on the generated tree. The tube function $T : R^3 \to R$ in Eq. (1) is a signed distance transformation that maps every voxel point $\mathbf{x}$ to the tube surface along the centerline [2] such that points inside the vessel have negative values, and positive outside the vessel with smooth transitions. This means a marching cube method can easily generate the surface mesh, and the binary label map can be easily generated by using a 0 threshold value,

$$T_{\mathbf{c},r}(\mathbf{x}) \equiv \min_{i} \left\{ \min_{\tau \in [0, L_i]} \left\{ \|\mathbf{x} - \mathbf{c_i}(\tau)\|^2 - r_i^2(\tau) \right\} \right\} \tag{1}$$

where $\mathbf{c_i}$ denotes the centerline graph of the ith line segment, L_i and r_i denote the length and radius of each vessel. τ denotes the exact arc length along the line segment starting at node position $\mathbf{s}_i$ and ending at node position $\mathbf{s}_{i+1}$. Hence, $\mathbf{c}_i(\tau) \equiv \mathbf{s}_{i+1}\left(\frac{\tau}{L_i}\right) + \mathbf{s}_i\left(1 - \frac{\tau}{L_i}\right)$ Assuming both the spatial coordinates and radii are linearly interpolated, τ can be found analytically by the expression for the closest point to a line in Euclidean geometry [2],

$$\tau \equiv \frac{(\mathbf{x} - \mathbf{c}_i(s_i)) \cdot (\mathbf{c_i}(s_{i+1}) - \mathbf{c_i}(s_i))}{\|\mathbf{c_i}(s_{i+1}) - \mathbf{c_i}(s_i)\|} \tag{2}$$

The process is extremely time-consuming because the tube function has to be evaluated over a large grid on each line segment (vessel) $[\mathbf{s}_i, \mathbf{s}_{i+1}]$ such that the tube function is minimized. A crucial acceleration we make here is that, for each line segment $[\mathbf{s}_i, \mathbf{s}_{i+1}]$, we only compute the tube function inside a bounding cube around it. Specifically, only voxel points within the bounding cube of $[\min(\mathbf{s}_i, \mathbf{s}_{i+1}) - \max(r(\mathbf{s}_i), r(\mathbf{s}_{i+1})), \max(\mathbf{s}_i, \mathbf{s}_{i+1}) + \max(r(\mathbf{s}_i), r(\mathbf{s}_{i+1}))]$ is evaluated for each line segment, where $\min(\mathbf{s}_i, \mathbf{s}_{i+1})$ and $\max(\mathbf{s}_i, \mathbf{s}_{i+1})$ are both in $\mathcal{R}^3$ representing the minimum and maximum coordinates of the two endpoints s_i and s_{i+1} in the three dimensions separately. This simple modification reduces the computational time from weeks to only one minute on a grid size of around $955 \times 508 \times 626$ over a generated vascular tree with around 50K vessel segments.

The corresponding scan image can be roughly created by adding some noise to the label maps based on micro-CT characteristics. Fig. 2 shows the example of a generated label map from the reconstructed tree by applying a threshold on the tub function as well as the synthesized image with some random Gaussian and Salt&Pepper noise. Pretraining a neural network for renal vessel segmentation would require more information on the scanning

details of the micro CT devices to emulate the imaging process and generate realistic noises, which is far beyond the scope of this work.

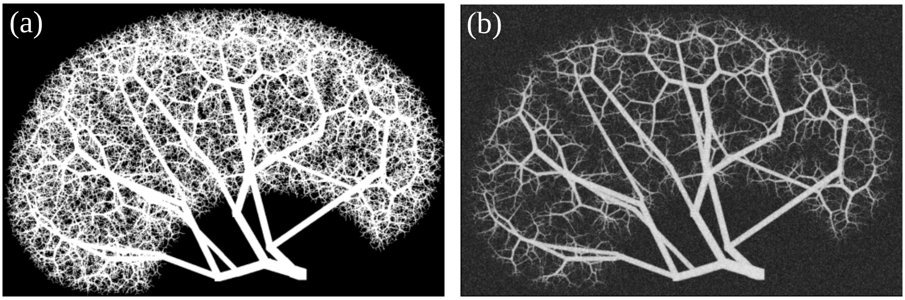

Fig. 2: Example of the maximum intensity projection of a generated vessel label map from the reconstructed tree (a) and the synthesized image with random Gaussian and Salt&Peper noise (b). Note that they are mapped back to the original voxel space of 22.6 μm, meaning that some small vessels are not visible.

3.1 Convergence plot

We finally show the convergence plot of the global cost function $C(\mathcal{G})$ over the whole GCO process in Fig. 3. Here the spikes indicate the pruning operations after each iteration, which removes deep branches. The pruning threshold is decreased after two iterations so that more branches are reserved. Although the decrease in cost is extremely subtle after each iteration of pruning, it gradually produces new trees with better global branching structures in the next iteration. Overall, the algorithm reaches convergence after several iterations with spikes due to pruning operations.

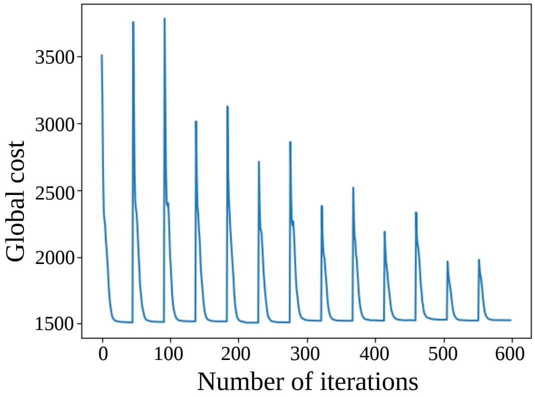

Fig. 3: Convergence plot over the GCO process.

References

[1] Ahrens, J., Geveci, B., Law, C.: Paraview: An end-user tool for large data visualization. The visualization handbook **717**(8) (2005)

[2] Antiga, L., Steinman, D.A.: Robust and objective decomposition and mapping of bifurcating vessels. IEEE transactions on medical imaging **23**(6), 704–713 (2004)
